# Allelic Variation, Alternative Splicing and Expression Analysis of *Psy1* Gene in *Hordeum chilense* Roem. et Schult

**DOI:** 10.1371/journal.pone.0019885

**Published:** 2011-05-16

**Authors:** Cristina Rodríguez-Suárez, Sergio G. Atienza, Fernando Pistón

**Affiliations:** Departamento de Mejora Genética Vegetal, Instituto de Agricultura Sostenible - Consejo Superior de Investigaciones Scientificas, (IAS-CSIC), Córdoba, Spain; University of Melbourne, Australia

## Abstract

**Background:**

The wild barley *Hordeum chilense* Roem. et Schult. is a valuable source of genes for increasing carotenoid content in wheat. Tritordeums, the amphiploids derived from durum or common wheat and *H. chilense*, systematically show higher values of yellow pigment colour and carotenoid content than durum wheat. Phytoene synthase 1 gene (*Psy1*) is considered a key step limiting the carotenoid biosynthesis, and the correlation of *Psy1* transcripts accumulation and endosperm carotenoid content has been demonstrated in the main grass species.

**Methodology/Principal findings:**

We analyze the variability of *Psy1* alleles in three lines of *H. chilense* (H1, H7 and H16) representing the three ecotypes described in this species. Moreover, we analyze *Psy1* expression in leaves and in two seed developing stages of H1 and H7, showing mRNA accumulation patterns similar to those of wheat. Finally, we identify thirty-six different transcripts forms originated by alternative splicing of the 5′ UTR and/or exons 1 to 5 of *Psy1* gene. Transcripts function is tested in a heterologous complementation assay, revealing that from the sixteen different predicted proteins only four types (those of 432, 370, 364 and 271 amino acids), are functional in the bacterial system.

**Conclusions/Significance:**

The large number of transcripts originated by alternative splicing of *Psy1*, and the coexistence of functional and non functional forms, suggest a fine regulation of PSY activity in *H. chilense*. This work is the first analysis of *H. chilense Psy1* gene and the results reported here are the bases for its potential use in carotenoid enhancement in durum wheat.

## Introduction

Carotenoids are a diverse family of isoprenoid derived pigments found in plants, fungi and bacteria. They are involved in many critical plant processes such as photosynthesis, abscisic acid synthesis or prevention of photo-oxidative damage [1]. Regarding human nutrition, carotenoids are precursors of vitamin A and other nutritional factors, having essential roles in human health as antioxidants. Therefore, in the last years, increasing carotenoid content in seed endosperm of the main crop staples (rice, maize and wheat) has been a breeding objective and great efforts have been conducted to better understand carotenoid biosynthetic pathways and regulation [2], [3].

The first step of the carotenoid pathway is catalyzed by the enzyme phytoene synthase (PSY) which is encoded by three paralogous genes in grasses [4]. From the three paralogs, *Psy1*, *Psy2* and *Psy3*, only *Psy1* is related to endosperm carotenoid content [4]–[6] being considered a rate-limiting step in carotenoid accumulation.

Commonly, carotenoid content has not been a direct target for breeding in wheat. Instead, yellow pigment content (YPC) has been generally considered. A bright yellow colour is required for durum wheat (*Triticum turgidum*) for pasta production and, therefore, durum wheat breeding programs worldwide have selected high YPC varieties.

QTLs for YPC have been identified in several chromosomes in both durum and bread wheat, being those located on the chromosomes from the homoeologous group 7 the main responsible of the variation. *Psy1* was considered a candidate gene to explain the high seed YPC of wheat grain since it plays a critical role in the carotenoid pathway and maps to chromosomes 7A and 7B of durum and bread wheat [7], [8]. From the first report confirming the association of *Psy-B1* with variation in YPC in durum wheat seeds [8], several works have demonstrated co-segregation of *Psy-A1* and *Psy-B1* with QTLs for YPC in both durum and bread wheat [9]–[12]. Additionally, the role of *Psy1* genes in YPC content has been also suggested using association studies and *Psy1* allele-specific markers of high and low YPC wheat varieties have been developed [10], [13].

The wild barley *Hordeum chilense* (Roem. et Schultz.) is a valuable source of genes for increasing carotenoid content. The high compatibility of *H. chilense* with the genomes of *Triticum* species give raise to fertile and stable amphiploids and allows the transfer of traits to wheat [14]. The species shows a wide range of variation (at both morphological and molecular levels) distributed into two main groups plus an intermediate group, as revealed by molecular markers [15], [16]. Hexaploid tritordeums (x*Tritordeum* Ascherson et Graebner) are the amphiploids derived from the cross between *H. chilense* and durum wheat. They exhibit a high YPC [17] and higher carotenoid content than their durum wheat parents (up to 8-fold) [18]. Using the series of addition lines of *H. chilense* into common wheat, it was shown that chromosome 7H^ch^ was the main responsible of the high carotenoid content in tritordeums [19]. A further work mapped *Psy1* gene of *H. chilense* to the α-arm of chromosome 7H^ch^ with a diagnostic CAP marker [7]. Therefore, *Psy1* gene of *H. chilense* is a good candidate for the improvement of YPC and has a great potential in durum wheat breeding. For this purpose, characterization of *Psy1* gene in *H. chilense* is required for an effective use in durum wheat breeding.

Accordingly, the objectives of this work were to characterize *Psy1* gene from *H. chilense* in four main aspects (1) allelic variability (2) transcript analysis (3) functional analysis of the transcripts and (4) expression analysis.

## Results

### Characterization of *Psy1* genomic sequence

Lines H1, H7 and H16 of *H. chilense* were selected for this work, as they represent the three ecotypes described for the species [15], [16]. The amplification of the *Psy1* 5′ and 3′ ends from cDNA synthesized from line H1 was carried out by RACE-PCR using the primer pairs included in Table 1. The identity of the amplified fragments was confirmed with BLASTn. The resulting fragments shared high homology with *Psy1* genes of different plant species. Considering the consensus sequence, new primers were designed to amplify both the complete genomic sequence and the cDNA coding sequence of *Psy1* gene.

**Table 1:** Primers used for the characterization of *Psy1* gene in *H. chilense*.

Primer	Purpose	Sequence 5′→3′
RvPsy1con1	5′ SP	GCCCTCTTGATCTGCCTCTTCATG
RvPsy1con3	5′ SP	CATATGGCGCGCCGCCGCTCCTC
	NP for RvPsy1con1	
RvPsy1con2	NP for RvPsy1con3	ACGTCGTACACCTTCTGCTCCGA
FwPsy1con2	3′ SP	TCGGAGCAGAAGGTGTACGACGT
FwPsy1con1	NP for FwPsy1con2 and FwPsy1con3	CATGAAGAGGCAGATCAAGAGGGC
FwPsy1con3	3′ SP	CGGCGCGCCATATGGGCCATCTA
FwPsy1Hc2	3′ SP	TGTCGGAGAAGATGCAAGAAGAGGA
FwPsy1Hc1	NP for FwPsy1Hch2	CATCTCAAAGGAGTCGTCACCGAAA
HcPsy1-CDS3F	cDNA amplification	CACCACTCCGGCCGAGACAAAG
	Genomic DNA amplification	
	Full-length DNA sequencing	
HcPsy1-CDS4F	cDNA amplificationPhysical mapping	CATAGTGGTGAATCCATCCCTTGC
HcPsy1-5ER	Genomic DNA amplification	GTCGTTTGCCTCGATCTCAT
HcPsy1-CDS4R	cDNA amplification	CCTGACCATCTTCATCTTGCTTTG
HcPsy1-2EF	Genomic DNA amplification	CTTGCTGATGACGGAGGAG
	Full-length DNA sequencing	
HcPsy1-CDS5R	cDNA amplification	TGTCTTCAAACGCACATGTTCTAGC
	Genomic DNA amplification	
	Full-length DNA sequencing	
HcPsy1-3EF	Full-length DNA sequencing	GCCCCTAGACATGCTCGAA
HcPsy1-2ER	Physical mapping	GCCGCTCCTCCGTCATCAG
HcPsy1-4EF	Full-length DNA sequencing	CTCGCGAACCAGCTCACC
HcPsy1RT-F1	RT-qPCR	CGAACCAGCTCACCAACATA
HcPsy1RT-R1	RT-qPCR	GAGCTCGTCTTGTGGCAAAT

SP: specific primer for RACE PCR; NP: nested primer for RACE PCR.

**Figure 1:**
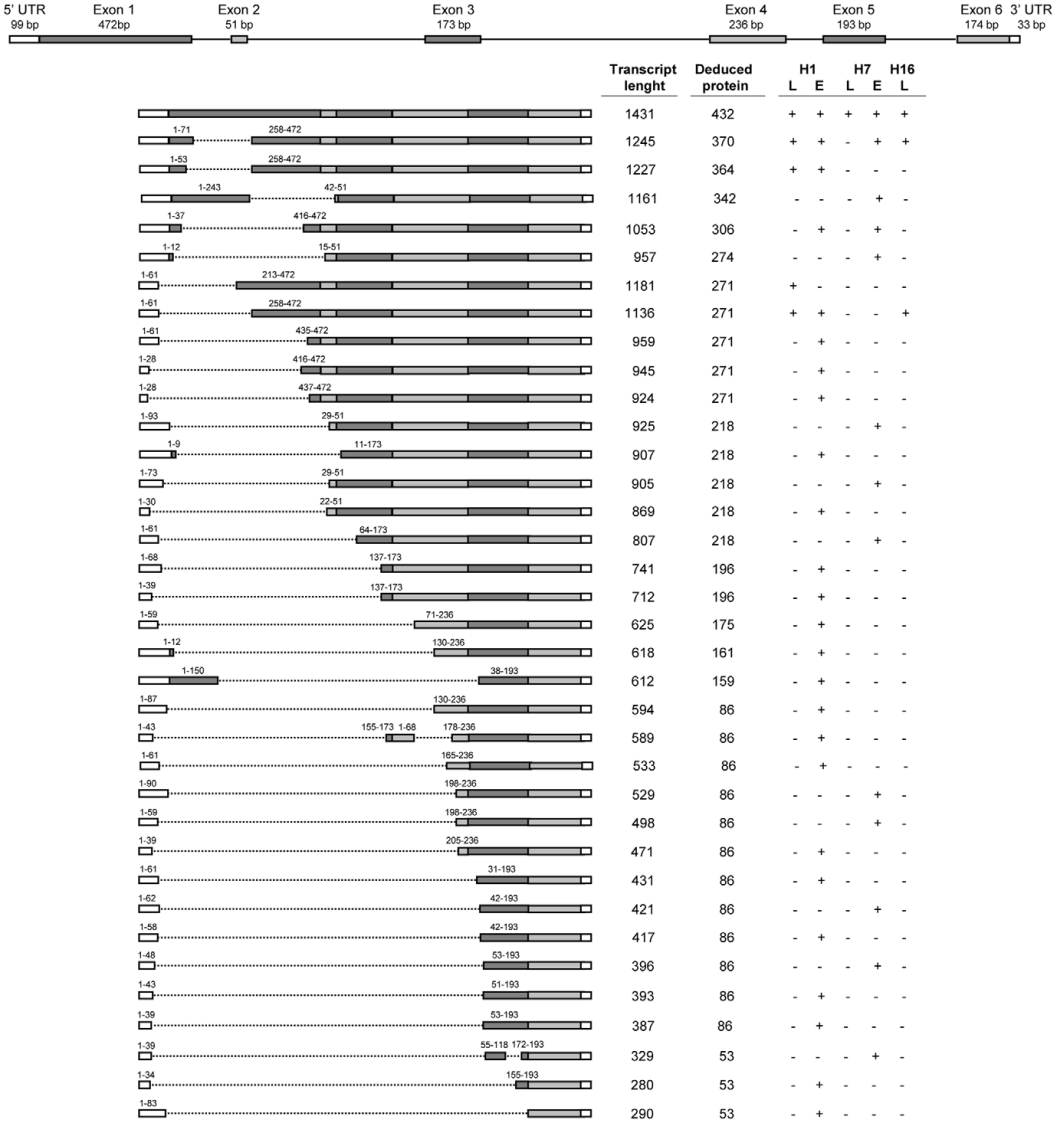
Schematic representation of *Psy1* splicing forms in *H. chilense*. Exons are represented by grey boxes and introns by a continuous line. White boxes represent 5′ and 3′ UTR. Discontinuous lines represent the untranscribed regions compared to the expected correct splicing form (1431 bp, 432 amino acids). For each transcript type, transcript length (bp), deduced protein length (amino acid residues) and the genotype/tissue where they were detected (L =  leaf; E =  endosperm; +  =  detected; -  =  not detected) is indicated. Exons length is indicated in the genomic form (top). When exons and/or 5′ UTR are not complete in a transcript form, it is indicated which part is included referring to the total exon and/or 5′ UTR length.

Using two primer pairs (HcPsy1-CDS3F/HcPsy1-5ER and HcPsy1-2EF/HcPsy1-CDS5R), two overlapping segments of *Psy1* were amplified, cloned and sequenced in H1, H7 and H16 genomic DNA. The genomic DNA sequence of *Psy1* contained 3,447 bp in H1; 3,400 bp in H7 and 3,396 bp in H16, including 173 and 265 bp of the 5′ UTR and 3′ UTR flanking regions, respectively (GenBank accession numbers HM598415, HM598416 and HM598417, respectively). Complete genomic sequence had a 97.3% homology between *Psy1* alleles of H1 and H7; 98.2% between H1 and H16 and 98.7% between H7 and H16. The exon/intron structure of *H. chilense Psy1* was inferred from the alignment of genomic sequence and the cDNA coding region and compared with its homologous from wheat (Figure 1). It consisted of six exons and five introns, being the exons of the same length in the three lines. The sequence of exons 2, 3 and 6 was identical in the three alleles and exons 1, 4 and 5 showed high sequence identities: 8 single nucleotide polymorphisms (SNPs) between H1-H7 and between H7-H16, and 2 SNPs between H1-H16. Otherwise, the introns differed in size among the three alleles, and showed a higher number of SNPs and insertion-deletion as expected for intronic regions. A 49 nucleotide insertion was identified in the intron 3 of H1, showing a partial inverted duplication of the sequences flanking the insertion.

The molecular phylogenetic tree was constructed using the alignment of *Psy1* coding sequences from *Oryza sativa*, *Zea mays*, *Triticum aestivum*, *Triticum turgidum*, *Triticum urartu*, *Triticum monococcum*, *Aegilops tauschii* and *Aegilops speltoides.* The *Psy2* sequences of *O. sativa and Z. mays*, two partial sequences of *Psy2* of *Triticum turgidum*, and *Psy3* sequences from *O. sativa* and *Z. mays* were used like out-group. The sequences from *H. chilense* obtained in this work were grouped with the *Psy1* sequences (Figure S1). Moreover, *Psy1* sequences from *H. chilense* are closest to the *Psy1* from wheat D genome by sharing a similarity of 97.5% (96.5% with A genome and 95.8% with B genome).

### Quantitative real-time PCR

Once we had *Psy1* sequence of *H. chilense*, we designed the primer pair HcPsy1RT-F1/HcPsy1RT-R1 to carry out a preliminary qPCR assay, in order to have an overview of the expression rates of *Psy1* in *H. chilense*. A fragment of 80 bp of *Psy1* gene located between exons 4 (197–236 bp) and 5 (1–40 bp) was amplified. The product obtained in the amplification of cDNA from H7 leaves (only one product expected) was sequenced. The identity of this fragment was confirmed by BLASTn (showing the highest homology with *Psy1* genes of different species) and by the alignment with *Psy1* sequences from *H. chilense* obtained in this work, confirming that the amplified fragment corresponds to the end of exon 4 and the beginning of exon 5, as expected.

**Figure 2:**
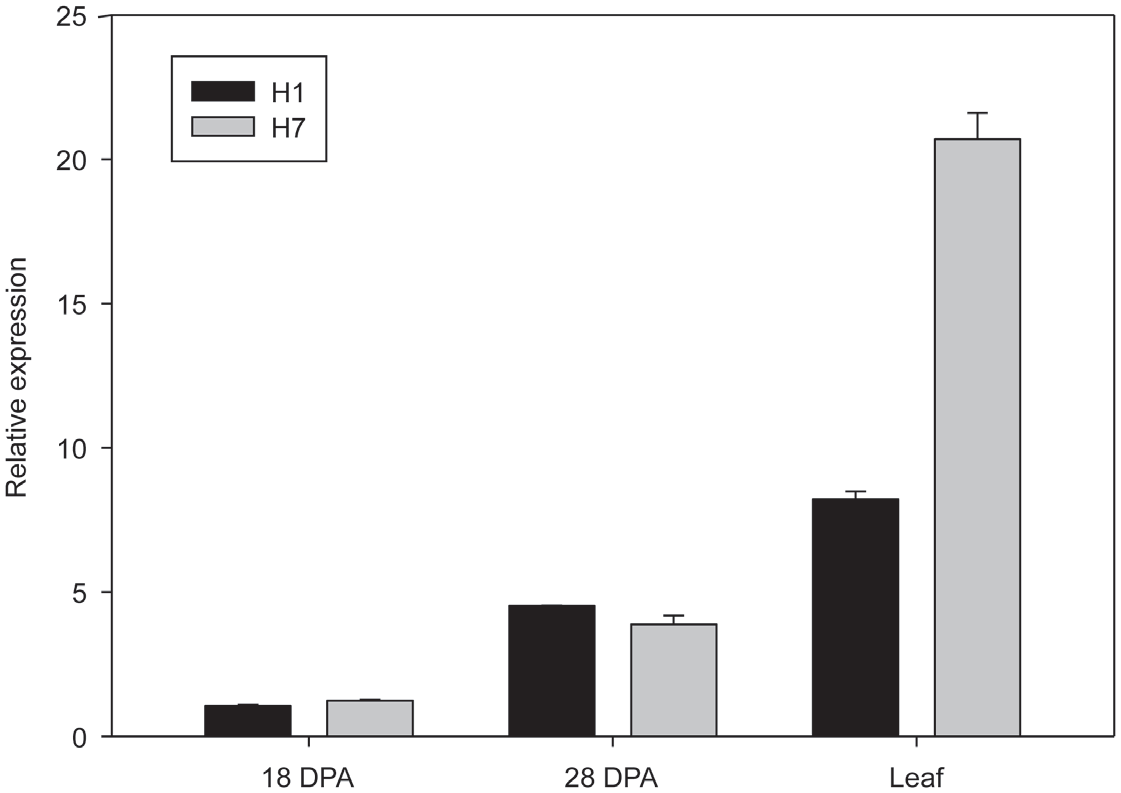
Transcript levels of *Psy1* in leaf and seeds in *H.chilense* lines H1 and H7. *Psy1* expression levels in seeds were measured at 18 and 28 days after anthesis (DPA) in both lines. Values in the Y axis are normalized and calibrated relative to reference genes. Error bars are SE of the means.

The expression of *Psy1* was measured by qPCR in leaves and in seeds at 18 and 28 DPA in lines H1 and H7 (Figure 2). The expression of *Psy1* was higher in leaves than in seeds, being 7.75 and 1.82 times higher in leaf than in seeds at 18 DPA and 28 DPA respectively in line H1, and 16.68 and 5.33 times higher in leaf than in seeds at 18 DPA and 28 DPA respectively in line H7. During seed developing, expression of *Psy1* was increased, showing the highest value at 28 DPA in both lines. The *Psy1* expression between lines H1 and H7 was only significantly different in leaf (LSD, *p*<0.05), being more than twice higher in H7.

### Characterization of *Psy1* transcripts

The full coding sequence of *Psy1* was amplified using primer pairs HcPsy1-CDS3F/HcPsy1-CDS5R (primer pair 1) or HcPsy1-CDS4F/HcPsy1-CDS4R (primer pair 2) in cDNA from leaves in lines H1, H7 and H16 and in two seed developing stages of H1 and H7 (at 18 and 28 DPA). Considering fragment sizes obtained with primer pair 2, a single product of 1431 bp was obtained in H7 leaves. However, more than one transcript was detected in H1 and H16 leaves and in endosperm samples from H1 and H7 lines. Regarding H1 leaves, we were able to identify five transcripts of 1431 bp, 1245 bp, 1227 bp, 1181 bp and 1136 bp. In H16 leaves, three PCR products of 1431 bp, 1245 bp and 1136 bp were obtained, being coincident with those found in H1 and H7.

Regarding endosperm, 25 different transcripts were detected in H1, being 21 of them exclusive (from tissue and genotype) and 4 of them the same as those of 1431 bp, 1245 bp, 1227 bp and 1136 bp found in H1 leaves. In H7 endosperm samples 13 transcripts were found, being 10 of them exclusive (from tissue and genotype), one in common with H1 endosperm (1053 bp) and 2 also found in H1 leaves and endosperm and H16 leaves (1431 bp and 1245 bp). Figure 1 describes all transcripts characterized in this work, indicating transcripts length (bp), deduced proteins length (aminoacid residues) and the genotype/tissue where they were detected. All cDNA sequences obtained in this work were deposited in the GenBank (accession numbers JF759623 to JF759664).

**Figure 3:**
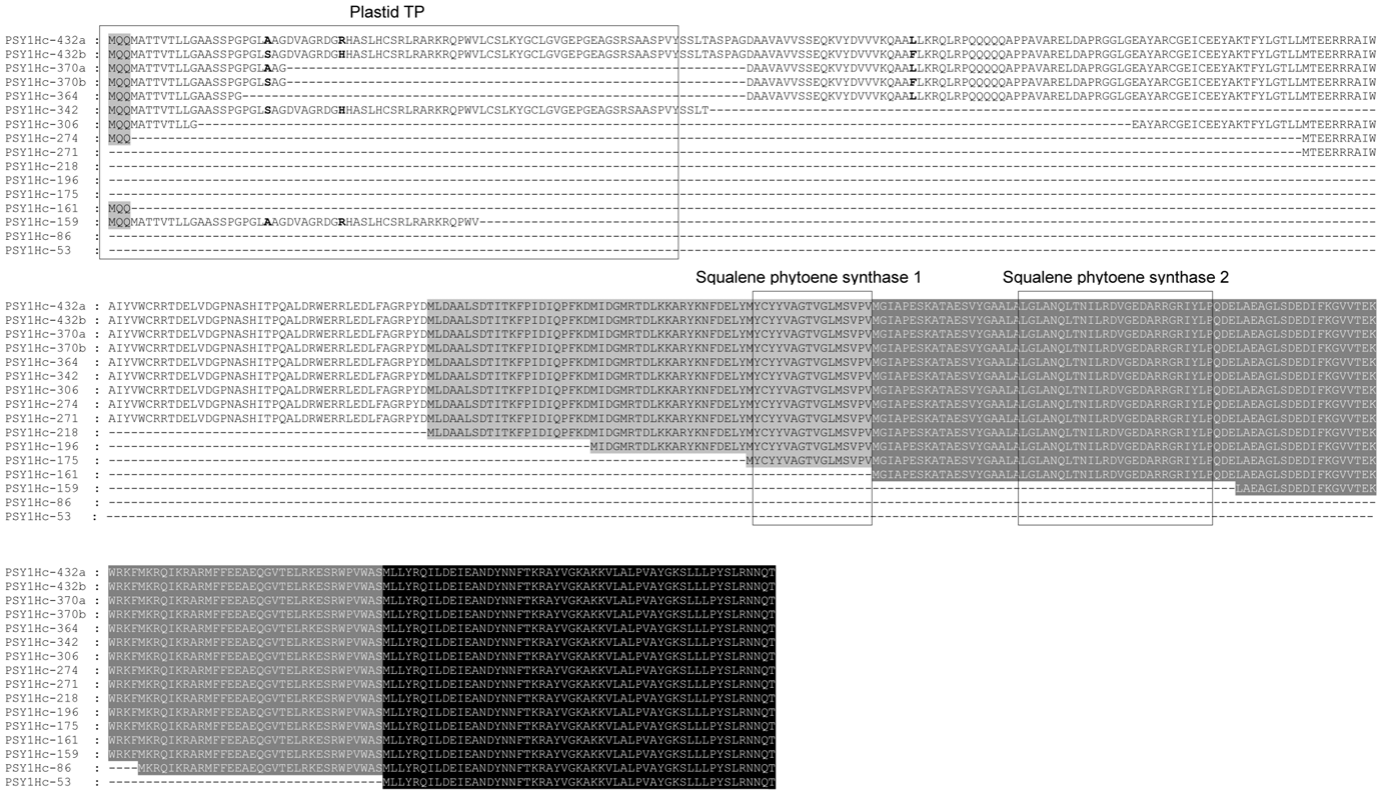
Alignment of the deduced amino acid sequences from the *Psy1* transcripts identified in *H. chilense*. The plastid transit peptide (TP) and squalene-phytoene synthase 1 and 2 domains are indicated. The sixteen proteins are named PSY1Hc- followed by the number of amino acid residues. If the protein type is polymorphic between lines it is indicated: (a) H1/H16 form and (b) H7 form. Polymorphic positions are in bold.

Alignment of the deduced PSY1 aminoacid sequences (those expected from the correctly spliced forms) revealed three residue differences in H7 when compared to H1 and H16, all them located in exon 1: an Ala to Ser substitution at position 22, an Arg to His substitution at position 32 and a Leu to Phe substitution at position 109. In total, the following 16 different proteins are deduced from the 36 transcript types detected (named PSY1Hc- followed by the number of amino acid residues): PSY1Hc-432a (in H1 and H16), PSY1Hc-432b (in H7), PSY1Hc-370a (in H1 and H16), PSY1Hc-370b (in H7), PSY1Hc-364, PSY1Hc-342, PSY1Hc-306, PSY1Hc-274, PSY1Hc-271, PSY1Hc-218, PSY1Hc-196, PSY1Hc-175, PSY1Hc-161, PSY1Hc-159, PSY1Hc-86 and PSY1Hc-53. Figure 3 shows the alignment of the sixteen deduced PSY1 proteins of *H. chilense*. Using the TargetP transit peptide (TP) predictor, the *H. chilense* PSY1 was predicted to have a plastid transit peptide. Exact length of *H. chilense* PSY1 TP was deduced from the alignment with the maize and *Arabidopsis thaliana* PSY1 protein sequences (AAR08445 and P37271, respectively). Thereby, *H. chilense* PSY1 was predicted to have a plastid TP of 77 amino acids from the N-terminal end (Figure 3). The complete TP sequence is present in PSY1Hc-432a, -432b and -342 deduced proteins; forms PSY1Hc-370a, -370b, -364, -306, -274, -161 and -159 show truncated TPs; and protein types PSY1Hc-271, -218, -196, -175, -83 and -53 do not show any region of TP (Figure 3).

### Functional analysis of *H. chilense Psy1* transcripts

To determine which transcripts were functional, transcripts coding for the sixteen different protein types were cloned and co-transformed with pAC-85b plasmid [20]. This plasmid contains all genes needed to produce β-carotene in *Escherichia coli* except for one encoding phytoene synthase. β-carotene production was detected by yellow color production of bacterial pellet and reversed-phase high-performance liquid chromatography (RP-HPLC) analysis of extracts from the co-transformed *E. coli* strains (Figure S2), showing that only PSY1Hc-432a, -432b, -370a, -364 and -271 proteins were functional in the bacterial system. All constructions produced similar rates β-carotene except for PSY1Hc-370a which produced a lower quantity (traces). Unfortunately, transcript type of 1245 bp detected in H7 endosperm and producing PSY1Hc-370b protein could not be tested after several attempts (see material and methods). Table 2 summarizes the results obtained in the functional analysis of *Psy1* transcripts.

**Table 2:** Functional analysis of *Psy1* transcripts of *H. chilense*.

		Function	
Protein type	Transcript tested	Color^1^	β-carotene^2^
PSY1Hc-432a	1431	Yellow	Presence
PSY1Hc-432b	1431	Yellow	Presence
PSY1Hc-370a	1245	Pale Yellow	Traces
PSY1Hc-370b	-	-	-
PSY1Hc-364	1227	Yellow	Presence
PSY1Hc-342	1161	White	Absence
PSY1Hc-306	1053	White	Absence
PSY1Hc-274	957	White	Absence
PSY1Hc-271	1136	Yellow	Presence
PSY1Hc-218	869	White	Absence
PSY1Hc-196	712, 741	White	Absence
PSY1Hc-175	625	White	Absence
PSY1Hc-161	618	White	Absence
PSY1Hc-159	612	White	Absence
PSY1Hc-86	533, 529, 471, 417, 396	White	Absence
PSY1Hc-53	329, 290	White	Absence

1 Color complementation observed in *E. coli* colonies co-transformed with pAc-85b plasmid and each of the expression vectors harboring *H. chilense Psy1* transcripts.

2 β-carotene production detected by HPLC in co-transformed *E. coli* pellets.

## Discussion

*Psy1* gene of *H. chilense* contains six exons and five introns, showing the same structure as in other related species previously described [21], [22]. Nevertheless, the first exon of *Psy1* gene in *H. chilense* is longer than *Psy1* genes of maize, rice and wheat, and the sixth exon has the same length as in maize and rice and it is longer than in wheat. Compared to the three *Psy1* genes in common wheat, *Psy1* sequence in *H. chilense* is more related to *Psy-D1* than to *Psy-A1* or *Psy-B1*. Consistently, cytological characterizations [23] and marker transferability analyses [15] have also suggested that *H. chilense* is more related to wheat D-genome than to A and B genomes.

Regarding *H. chilense* intraspecific nucleotide variability, most of the polymorphisms are found in the intronic regions, as expected. SNPs are also found in exons one, four and five, but only three of them, located in exon one, lead to aminoacid changes in line H7 compared to H1 and H16.

The surprising abundance of different transcripts in *H. chilense* raises several questions. First, the possible mixture of transcripts of different *Psy* genes could be considered. In grasses, three paralogs (*Psy1*, *Psy2* and *Psy3*) originated from duplication events, have been described [4], [24], [25]. The *Psy* sequences identified in this work are unequivocally included in the cluster comprising *Psy1* sequences of wheat, maize and rice (Figure S1). Furthermore, when amplifying *Psy1* using primer pair HcPsy1-CDS4F/HcPsy1-2ER in the set of addition lines of *H. chilense* in common wheat, a positive amplification is only observed in lines carrying the 7H^ch^ complete chromosome or the α-arm of chromosome 7H^ch^ (Figure S3). *Psy1* has been located in this chromosomal position [7] whereas *Psy2* and *Psy3* are located in short and long arms of wheat chromosome 5 respectively, inferred by direct mapping or by synteny with rice chromosomes [4], [7], [26]. Additionally, a partial genomic sequence of *Psy2* gene of *H. chilense* had been obtained in a previous work [7]. Once aligned with the equivalent region of *Psy1* genomic and cDNA sequences of *H. chilense*, and considering the coding region, *Psy1* and *Psy2* have a sequence homology of only 74.5% (Figure S4). Thus, if any *Psy2* form had been amplified, it could have been easily recognized. The same happens with *Psy3* gene. Although *Psy3* has not been characterized in many species, a putative sequence of *Hordeum vulgare* is available in GenBank as well as a putative *Psy1* sequence (AK365521.1 and AK374031.1, respectively). When aligned with *Psy1* from *H. chilense* there is no doubt of the identity of each of the sequences (Figure S5). Finally, the different *Psy* splicing forms described in this work have identical sequence, differences are found in sequence length and therefore, the most plausible hypothesis is that they correspond to the same gene. For all these reasons, we can conclude that the occurrence of *Psy1*, *Psy2* and *Psy3* transcripts mixture seems unlikely.

Another hypothesis would be the presence of an additional *Psy* duplication located in the α-arm of chromosome 7H^ch^. There are evidences of an additional locus in the long arm of chromosome 7A involved in YPC in durum wheat [11], the possibility of being a new *Psy*-like sequence cannot be discarded, although gene duplication cannot explain the abundance of different *Psy1* transcripts in *H. chilense*.

On the other hand, it cannot be ruled out the possibility of that some of the transcripts considered as from endosperm come indeed from the embryo or other parts of the grain, as the small size of *H. chilense* seeds makes technically difficult to isolate it. Anyway, the abundance of different transcripts for *Psy1* gene in *H. chilense* is evident and intriguing.

Alternative splicing in plants is an important post-transcriptional regulatory mechanism. This phenomenon results in gain or loss of protein function, changes in cellular localization, stability and activity among others [27]. In rice, alternative splicing forms of *Psy1* gene have been described, resulting in four different transcripts (LOC_Os06g51290; http://rice.plantbiology.msu.edu). Besides, in *Psy-A1* of wheat, Howitt et al. [9] identified a sequence duplication in exon 2 that creates a new splicing site and activates a cryptic exon. This alternative splicing produced four different transcripts: the wild type, two shorter transcripts originated by frame-shift mutations and early terminations, and a fourth transcript 35 aminoacids longer than the wild type. The same transcripts were observed in leaves and endosperm, and only the wild type produced an active protein. In our work, we identified 36 different *Psy1* transcripts in *H. chilense*, being the largest spliced form (1431 bp) the size expected for correct splicing, and producing a protein of 432 amino acids. The rest of the forms are the result of different splicing events occurring in exons 1 to 5 and/or in the 5′ UTR and producing shorter proteins (Figure 1). Functionality of the predicted proteins was tested in a color complementation assay showing that only some of the transcripts lead to functional proteins in the bacterial system. As expected, both PSY1Hc-432a and -432b (predicted correctly spliced forms) complement the absence of *crtB* (bacterial phytoene synthase) gene in plasmid pAc-85b, producing β-carotene. The same happens with proteins PSY1Hc-370a, PSY1Hc-364 and PSY1Hc-271. Regarding protein PSY1Hc-370b, it is expectable to be a functional form as there is a single amino acid difference compared to the functional protein PSY1Hc-370a (an Ala to Ser substitution at position 22) and considering that both PSY1Hc-432a and PSY1Hc-432b are functional proteins despite their three amino acid differences, including that in position 22.

Several works have analyzed the activity of truncated forms of PSY proteins. Misawa et al. [28] demonstrated that tomato PSY proteins in which 114 amino acids from the N-terminal were eliminated had increased catalytic activity in *E. coli* compared to the complete form. In *Narcissus*, the full-length PSY enzyme showed reduced catalytic activity when compared with an N-truncated form with its transit sequence of 214 bp removed by site-directed mutagenesis [29]. More recently, in *Citrus*, the catalytic activity of PSY and a truncated form lacking 89 amino acids in its N-terminus was similar when analyzed their expression in *E. coli* cells [30]. *Psy1* is a nuclear encoded gene that mediates the first step in the plastid-localized carotenoid biosynthetic pathway. A large protein complex containing enzymes from the isoprenoid pathway and carotenogenic enzymes as PSY has been hypothesized to occur in the plastid stroma [31]. It is well known that transit peptides direct proteins to specific organelles. Consequently, although PSY1Hc-370a, PSY1Hc-364 and PSY1Hc-271 proteins are functional in the bacterial system, their implication in carotenoid synthesis or accumulation could be questioned, as their transit peptide is truncated or totally absent.

Subfunctionalization of the three *Psy* genes in grasses is accepted as a mechanism providing a fine control of carotenogenesis in response to developmental and environmental signals [6]. The surprising abundance of *Psy1* transcripts in *H. chilense*, only some of them leading to functional proteins, suggests a very accurate regulation of PSY1 active protein concentration and it would be possible to hypothesize that splicing to mature or alternative transcripts could also regulate the cellular concentrations of phytoene synthase, depending on the tissue, physiological or environmental conditions [9], [32].

*Psy1* expression was analyzed in leaves and in seeds at 18 and 28 DPA in lines H1 and H7. *Psy1* mRNA levels in leaves were higher than in seeds in both H1 and H7. Similar results were observed in common wheat, where *Psy1* mRNA was about 50-fold lower in immature milky stage endosperm samples than in leaves [33]. High abundance of *Psy1* transcripts has been also reported in maize leaves where they play an essential role in maintaining leaf carotenoid content, especially under heat stress conditions [5], [6]. Also in rice, PSY1 is the main enzyme responsible for carotenoid supply in chloroplasts [25]. Carotenoids are involved in photosynthetic processes like light harvesting and protection of the photosynthetic apparatus from photo-oxidation [34]. Regarding endosperm, in maize and rice it has been widely demonstrated the correlation of *Psy1* transcript accumulation and endosperm carotenoid content [5], [24], [35]. The association of allelic variation of *Psy1* with changes in grain pigment content has been also confirmed in both durum and bread wheat [8], [11], [36]. *Psy1* mRNA levels are not significantly different between lines H1 and H7 in the two seed development stages analyzed, although differences in yellow pigment content have been previously described in mature seeds [37]. Assuming that in *H. chilense* the correlation *Psy* mRNA accumulation-carotenoid content is also valid, the absence of association between YPC and mRNA levels can be explained. Alvarez et al. [37] determined values of 27.2 and 62.0 µg/g of carotenoid in H1 and H7 seeds, respectively. But actually, these measures refer to YPC as inferred by spectrophotometric determinations after water-saturated butanol extraction method, as it has been usually accepted in wheat. Carotenoids represent about 30–50% of the YPC in wheat [38], [39], but there are other factors also influencing final flour colour [40], [41]. On the other hand, *Psy1* mRNA levels are not in this case a direct estimation of functional PSY1 protein. In the pool of transcripts detected by qPCR only a part lead to functional proteins (Table 2). Additionally, 10 out of the 36 transcript types do not amplify with the selected primers (612 bp and 471–280 bp), although none of these transcripts have demonstrated to encode functional PSY1.

Moreover, other genes of the carotenoid synthesis pathway influence final carotenoid content and are also considered limiting steps, as it has been extensively studied in maize [35]. As an example, in the H1xH7 F_2_ mapping population, a QTL for YPC explaining the 14.8% of the phenotypic variance was identified in chromosome 2H^ch^ [42]. The carotenogenic gene ζ-carotene desaturase (ZDS) has been located in durum wheat chromosomes 2A and 2B [43] and in *H. chilense* chromosome 2H^ch^ (unpublished results), and it has been also associated with carotenoid accumulation in maize [44]. No QTLs for YPC were detected on chromosome 7H^ch^, probably due to the lack of polymorphism or saturation of this map region.

The characterization of *H. chilense Psy1* means the first step in the potential use of this gene for increasing carotenoid content in wheat breeding. First, we describe the allelic variability found among the three ecotypes at the genomic level, and we focus in H1 and H7 lines for the following works as they represent two distant groups considering their phylogenetic relations and YPC content. Regarding these two lines, we show the first results of *Psy1* expression in *H. chilense* and we evidence the surprising abundance of different *Psy1* transcripts forms, not ever described for this gene in other species. Finally, functional analysis of the splicing forms has been addressed, showing that only some transcript forms produce functional proteins and suggesting a fine modulation of functional PSY1 concentration.

As shown in Golden Rice 2, a variety of rice engineered to produce β-carotene, the use of new *Psy1* genes sources offers the potential to increase carotenoid content [45]. Accordingly, our results constitute an interesting basis for carotenoid enhancement in durum wheat either by chromosome engineering or by transgenesis. Functional analysis of *Psy1* will be useful for future selection of the appropriate transcripts sequences to increase the PSY1 activity in wheat by transformation. Moreover, the development of substitution lines of chromosome 7H^ch^ in durum wheat has been already started in order to test the effect of this chromosome in seeds carotenoid content.

## Materials and Methods

### Plant material

Accession lines of H1, H7 and H16 from the collection of the Instituto de Agricultura Sostenible, IAS-CSIC, Córdoba, were selected. Each line belongs to one of the three ecotypes described in *H. chilense* [16]. Leaf tissue of H1, H7 and H16 was harvested at tillering stage, frozen in liquid nitrogen, and stored at −80°C. Developing grains of H1 and H7 were harvested 18 and 28 DPA and conserved as described above. DNA from addition lines of *H. chilense* (line H1) into common wheat cv. Chinese Spring was used to confirm the location of the *Psy* fragment amplified.

### Amplification of cDNA ends

Total RNA from H1, H7 and H16 leaves was isolated using the TRIzol® Reagent (Invitrogen, Carlsbad, CA) according to manufacturer's instructions. Total RNA from H1 was used to synthesize anchored cDNA at the 5′ and 3′ ends as described by the SMART™ RACE cDNA Amplification Kit (Clontech, Palo Alto, CA). The specific primers were designed in the conserved regions of *Psy1* considering the complete coding sequences of maize (AY324431.1), rice (AY445521.1), common wheat A1b and D1a alleles (EF600064.1 and EU650397.1, respectively), durum wheat B1g allele (EU650396.1) and partial sequences of H1 and H7 lines of *H. chilense* (DU796677 and DU796679, respectively). All primers used in this work are included in Table 1.

RACE PCR reactions were carried out using Certamp complex enzyme mix (Biotools B&M Labs, Madrid) according to supplier's instructions and performed as follows: 25 cycles of 30 s at 94°C, 30 s at 64°C and 2 min at 72°C. PCR 5′ and 3′ products were reamplified using nested primers (see Table 1) and resolved on 1% agarose gels, stained with ethidium bromide and visualized under UV light. The resulting products were cloned in pGEMT-Easy vector (Promega, Madison, WI), and introduced into competent *Escherichia coli* (DH5*α*) cells by transformation. Plasmids were isolated and purified using Illustra plasmidPrep Mini Spin Kit (GE Healthcare, UK Ltd, UK) and used as template for sequencing.

### Amplification of CDS and full-length genomic sequence

The sequences obtained from 5′ and 3′ ends were used for primer design to amplify the complete coding sequence of *Psy1* in *H. chilense*. Total RNA was isolated from seeds at 18 and 28 DPA in lines H1 and H7 and from leaves of lines H1, H7 and H16 using the TRIzol® Reagent (Invitrogen, Carlsbad, CA) according to manufacturer's instructions. For the synthesis of first-strand cDNA, 4 µg of total RNA was reverse transcribed using oligo (dT) primer and M-MLV reverse transcriptase (Invitrogen, Carlsbad, CA) in 20 µl total volume according to the manufacturer's instructions. cDNA samples were diluted with additional 230 µl of water. cDNA PCR amplifications with primer combinations HcPsy1-CDS3F/HcPsy1-CDS5R (primer pair 1) or HcPsy1-CDS4F/HcPsy1-CDS4R (primer pair 2) were performed in 25 µl reactions consisting of 0.625 units of DNA polymerase (Biotools B&M Labs, Madrid), 1X PCR buffer, 2 mM MgCl_2_, 320 µM dNTPs (Promega, Madison, WI, USA), 1 M betaine (Sigma-Aldrich, St. Louis, MO), 0.6 µM of each primer and 1.2 µl of the cDNA dilution. PCR were carried out as follows: 5 min at 94°C, 28 cycles of 15 s at 94°C, 30 s at an annealing temperature progressively lowered from 68°C to 54°C by 0.5°C every cycle and 5 min at 72°C, 25 cycles of 15 s at 94°C, 30 s at 56°C and 5 min at 72°C, followed by 10 min at 72°C. Full-length *Psy1* cDNA sequences were obtained by cloning and sequencing the obtained PCR fragments as specified before. For transcripts description we will refer to fragment sizes obtained by amplification with primer pair 2.

Genomic DNA was extracted from leaves using CTAB method according to [46]. Genomic DNA sequences of H1, H7 and H16 were obtained by amplifying two overlapping fragments using primer combinations HcPsy1-CDS3F/HcPsy1-5ER and HcPsy1-2EF/HcPsy1-CDS5R. PCR amplification, cloning and sequencing was performed as described above for cDNA. *Psy1* full-length genomic sequences of H1, H7 and H16 were constructed by assembling the sequencing products obtained with primers HcPsy1-2EF, HcPsy1-3EF, HcPsy1-4EF, HcPsy1-CDS3F, HcPsy1-CDS5R (Table 1) and PSY1Rev2 [7]. In all cases, fragments were amplified twice to avoid artifacts in the sequencing stage.

### Functional analyses of *H. chilense Psy1* transcripts

The phytoene synthase gene from *Adonis aestivalis,* cloned into plasmid pAd-Psy [20], was cut and cloned into pGEMT-Easy vector to have a positive control of our co-transformation assays. It was noted that the insert was not expressed under T7 promoter and that only under SP6 promoter complementation was observed. All *Psy1* transcripts had been cloned under T7 promoter, thus, the vectors harboring the transcripts selected for co-transformations were cut with EcoRI, filled with A-tailing reaction, cloned again into pGEMT-Easy vector and selected for the right orientation (under SP6). Unfortunately, we could not recover any plasmid harboring the transcript leading to PSY1Hc-370b under SP6 promoter after several unsuccessful attempts.

For color complementation, TOP10F' competent cells (Invitrogen, Carlsbad, CA) were transformed with pAC85-b [20] and each one of the expression constructs with *Psy1* transcripts from *H. chilense*. Double transformants selected were grown overnight at 30°C in liquid LB medium containing ampicillin (150 mg/l), chloramphenicol (30 mg/l) and IPTG (50 mg/ml).

Cultured cells were harvested by centrifugation at 10,000×g, and the bacterial cell pellets were extracted with 3 ml of acetone containing 0.1% butylated hidroxy toluene (w/v), by the aid of sonication. The cell debris was separated by centrifugation at 5000 rpm at 4°C and the upper phase was dried under a nitrogen stream. The resulting residue was dissolved in 0.5 ml of acetone, centrifuged at 12,000×g and stored at −30°C until analyzed. Analysis of the carotenoid pigments was carried out by RP-HPLC as described by [47] with slight modifications.

### Quantitative real time PCR (qPCR)

For qPCR, the FastStart Universal SYBR Green Master (Roche Applied Science, Mannheim, Germany) was used on the 7500 Real Time PCR System (Applied Biosystems, Foster City, USA) together with the gene-specific primers HcPsy1RT-F1 and HcPsy1RT-R1 (Table 1) and the reference genes ADP-RF and RLI [48]. The PCR conditions were 95°C for 10 min followed by 40 cycles of 95°C for 15 s and 60°C for 1 min. All reactions were replicated twice.

PCR efficiency of each primer pair was determined with the LinRegPCR (version 11.0) quantitative PCR data analysis program [49] using raw normalized fluorescence as input data. Expression of the genes for each sample (N_0_) was determined by using the equation N_0_ = 0.2/E^Cq^, where E = PCR efficiency for each primer and Cq is the number of cycles needed to reach 0.2 arbitrary units of fluorescence. Levels of *Psy1* expression were normalized relative to the geometric mean of the reference genes ADP-RF and RLI. The significance of the differences in transcript level between samples was determined using ANOVA and a subsequent means comparison with Least Significant Difference method (LSD). Statistical methods were performed using the Rcmdr package of R [50], [51].

### Bioinformatic analyses

All primers were designed using Primer3plus software [52] (http://www.bioinformatics.nl/cgi-bin/primer3plus/primer3plus.cgi). Nucleotide and the deduced amino acid sequences were aligned using Edialign program (http://emboss.sourceforge.net/index.html) and edited using GeneDoc software (http://www.psc.edu/biomed/genedoc). Sequence identity searches were performed at the NCBI (http://www.ncbi.nlm.nih.gov) using BLAST.

The presence of a transit peptide was predicted by TargetP 1.1 Server (http://www.cbs.dtu.dk/services/TargetP/; [53]). The domains were predicted using InterProScan software (http://www.ebi.ac.uk/Tools/InterProScan/).

The coding sequence of *Psy1* alleles of lines H1, H7 and H16, along with some *Psy1* alleles previously described in wheat [11], [22], [36], [54], [55], partial sequences of wheat *Psy2* and the coding sequences of *Psy1*, *Psy2* and *Psy3* of maize and rice, were used to construct the phylogenetic tree. Neighbor joining tree was generated using the complete deletion method in MEGA4 program [56], [57]. Bootstrap test was performed with 1000 replicates to calculate the confidence of the different nodes.

## Supporting Information

Figure S1 **Neighbor-joining tree generated from the alignment of the** ***Psy1*** **coding sequences from different species.** Neighbor-joining tree generated from the alignment of the *Psy1* coding sequences from *Oryza sativa*, *Zea mays*, *Triticum aestivum*, *Triticum turgidum*, *Triticum urartu*, *Triticum monococcum, Aegilops tauschii* and *Aegilops speltoides.* The *Psy2* coding sequences of *O. sativa* and *Z. mays*, two partial sequences of *Psy2* of *Triticum turgidum*, and *Psy3* sequences from *O. sativa* and *Z. mays* were used like out-group. Predicted correctly spliced forms of *Psy1* gene in *H. chilense* lines H1, H7 and H16 were used. Numbers over the tree nodes are bootstrap confidence values based on 1,000 bootstrap iterations.
(TIF)

Figure S2 **Color complementation in co-transformed** ***E. coli*** **pellets and the corresponding RP-HPLC chromatograms of the carotenoid extracts.** (A) A yellow color is observed in *E. coli* pellets co-transformed with plasmid pAC-85b and pGEMT expressing proteins PSY1Hc-432a and PSY1Hc-432b, while no color complementation is observed when proteins PSY1Hc-86 or PSY1Hc-53 are expressed; (B) Absorption spectra in the HPLC mobile phase of β-carotene; (C) Corresponding RP-HPLC chromatograms of carotenoid extracts shown in (A).
(TIF)

Figure S3 **Chromosomal localization of** ***Psy1*** **gene in** ***H. chilense*** **.** Amplification of genomic DNA with primer pair HcPsy1-CDS4F/HcPsy1-2ER in the following lines: (1) *H. chilense* line H1, (2) *H. chilense* line H7, (3) *T. turgidum* ssp. *durum* cv. Yavaros, (4) *T. aestivum* cv. Chinese Spring, (5) *T. turgidum* var. Kofa, (6) *T. turgidum* breeding line UC1113, (7) 1H^ch^S monotelosomic addition line, (8) 1H^ch^S ditelosomic addition line, (9) 2H^ch^-α ditelosomic addition line, (10) 4H^ch^ disomic addition line (11) 5H^ch^ disomic addition line, (12) 5H^ch^L ditelosomic addition line, (13) 6H^ch^ disomic addition line, (14) 7H^ch^ disomic addition line, (15) 7H^ch^-α ditelosomic addition line, (16) 7H^ch^-β ditelosomic addition line and (17) 6H^ch^-S ditelosomic addition line.
(TIF)

Figure S4 **Alignment of partial** ***Psy1*** **and** ***Psy2*** **sequences in H1 and H7 lines of** ***H. chilense***. Alignment of genomic (g) and cDNA (c) sequences of *Psy1* (this work) and partial genomic sequence of *Psy2* gene in H1 and H7 lines (DU796678 and DU796680, respectively).
(TIF)

Figure S5 **Alignment of** ***Psy3*** **gene from different species**. Alignment of *Psy3* sequences of *Sorghum bicolor* (Sb; AY705390.1), *Zea mays* (Zm; NM_001114653.1), *Oryza sativa* (Os; FJ214953.1) and *Hordeum vulgare* clone NIASHv2034M16 showing high homology with *Psy3* gene of other species (AK365521.1), *Hordeum vulgare* clone NIASHv3051L01 showing high homology with *Psy1* gene of other species (AK374031.1) and *H. chilense Psy1* gene (this work).
(TIF)
