# Supplementary figures and images for: Allelic Variation, Alternative Splicing and Expression Analysis of Psy1 Gene in Hordeum chilense Roem. et Schult
Source: PLoS One. 2011 May 16;6(5):e19885. doi: 10.1371/journal.pone.0019885 (PMC3095628; doi:10.1371/journal.pone.0019885)

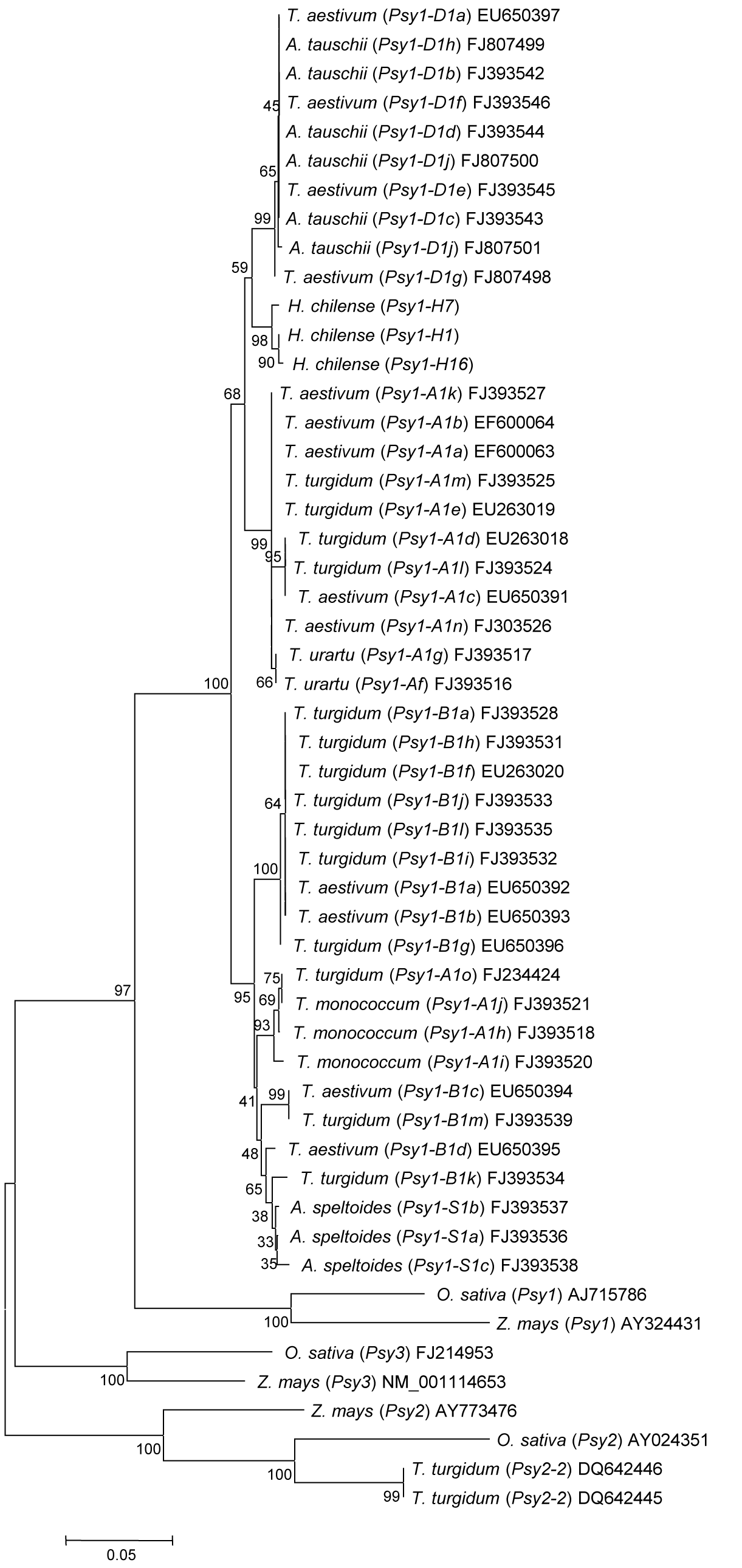

Supplement: Figure S1 — Neighbor-joining tree generated from the alignment of the Psy1 coding sequences from different species. Neighbor-joining tree generated from the alignment of the Psy1 coding sequences from Oryza sativa, Zea mays, Triticum aestivum, Triticum turgidum, Triticum urartu, Triticum monococcum, Aegilops tauschii and Aegilops speltoides. The Psy2 coding sequences of O. sativa and Z. mays, two partial sequences of Psy2 of Triticum turgidum, and Psy3 sequences from O. sativa and Z. mays were used like out-group. Predicted correctly spliced forms of Psy1 gene in H. chilense lines H1, H7 and H16 were used. Numbers over the tree nodes are bootstrap confidence values based on 1,000 bootstrap iterations. (TIF) [file pone.0019885.s001.tif]

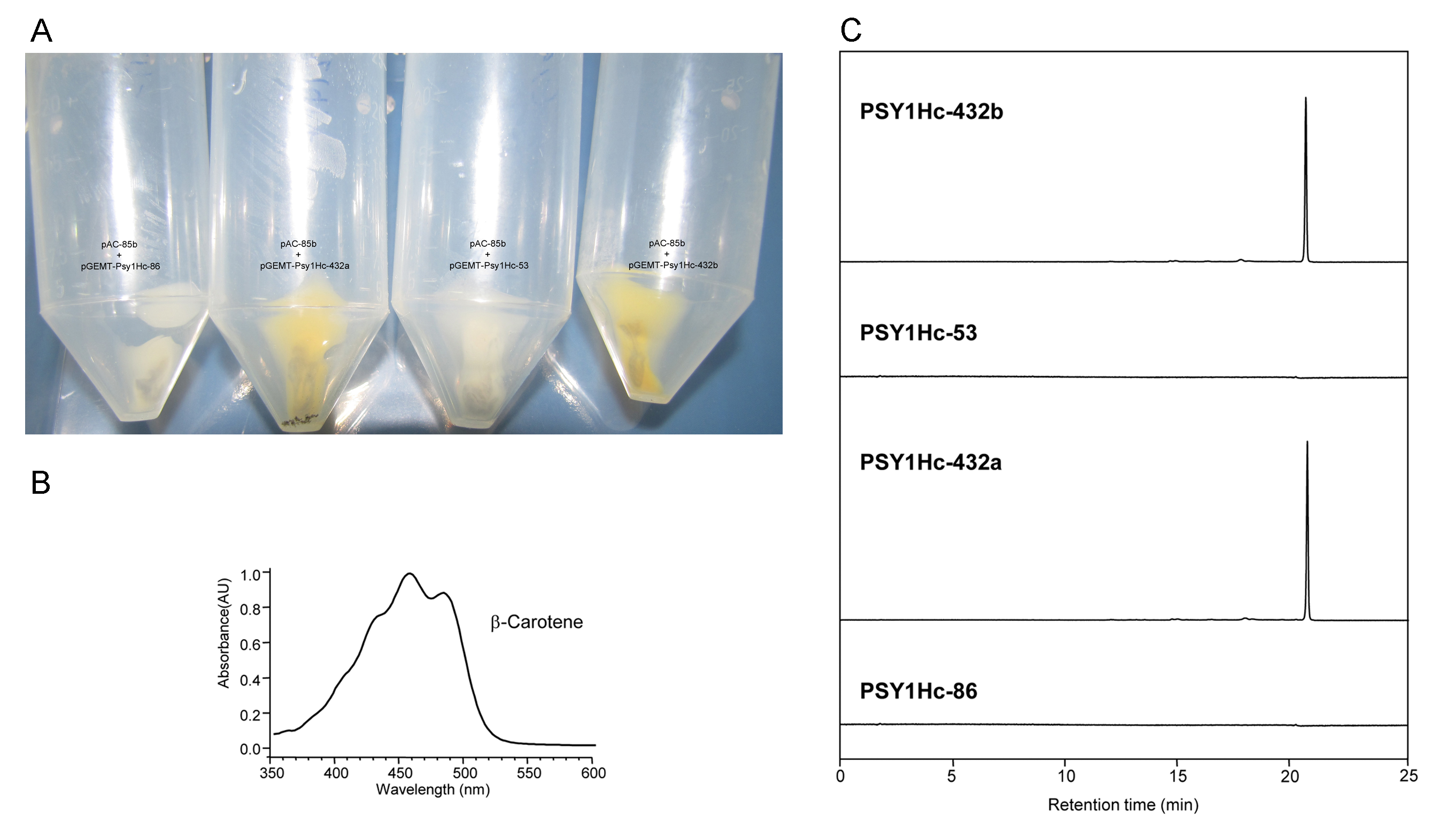

Supplement: Figure S2 — Color complementation in co-transformed E. coli pellets and the corresponding RP-HPLC chromatograms of the carotenoid extracts. (A) A yellow color is observed in E. coli pellets co-transformed with plasmid pAC-85b and pGEMT expressing proteins PSY1Hc-432a and PSY1Hc-432b, while no color complementation is observed when proteins PSY1Hc-86 or PSY1Hc-53 are expressed; (B) Absorption spectra in the HPLC mobile phase of β-carotene; (C) Corresponding RP-HPLC chromatograms of carotenoid extracts shown in (A). (TIF) [file pone.0019885.s002.tif]

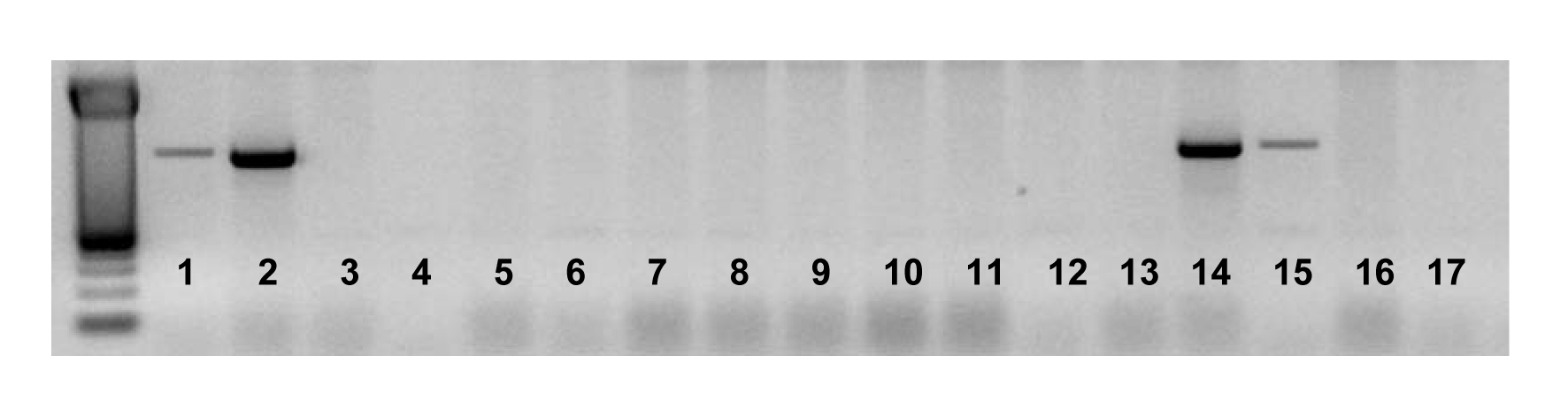

Supplement: Figure S3 — Chromosomal localization of Psy1 gene in H. chilense . Amplification of genomic DNA with primer pair HcPsy1-CDS4F/HcPsy1-2ER in the following lines: (1) H. chilense line H1, (2) H. chilense line H7, (3) T. turgidum ssp. durum cv. Yavaros, (4) T. aestivum cv. Chinese Spring, (5) T. turgidum var. Kofa, (6) T. turgidum breeding line UC1113, (7) 1HchS monotelosomic addition line, (8) 1HchS ditelosomic addition line, (9) 2Hch-α ditelosomic addition line, (10) 4Hch disomic addition line (11) 5Hch disomic addition line, (12) 5HchL ditelosomic addition line, (13) 6Hch disomic addition line, (14) 7Hch disomic addition line, (15) 7Hch-α ditelosomic addition line, (16) 7Hch-β ditelosomic addition line and (17) 6Hch-S ditelosomic addition line. (TIF) [file pone.0019885.s003.tif]

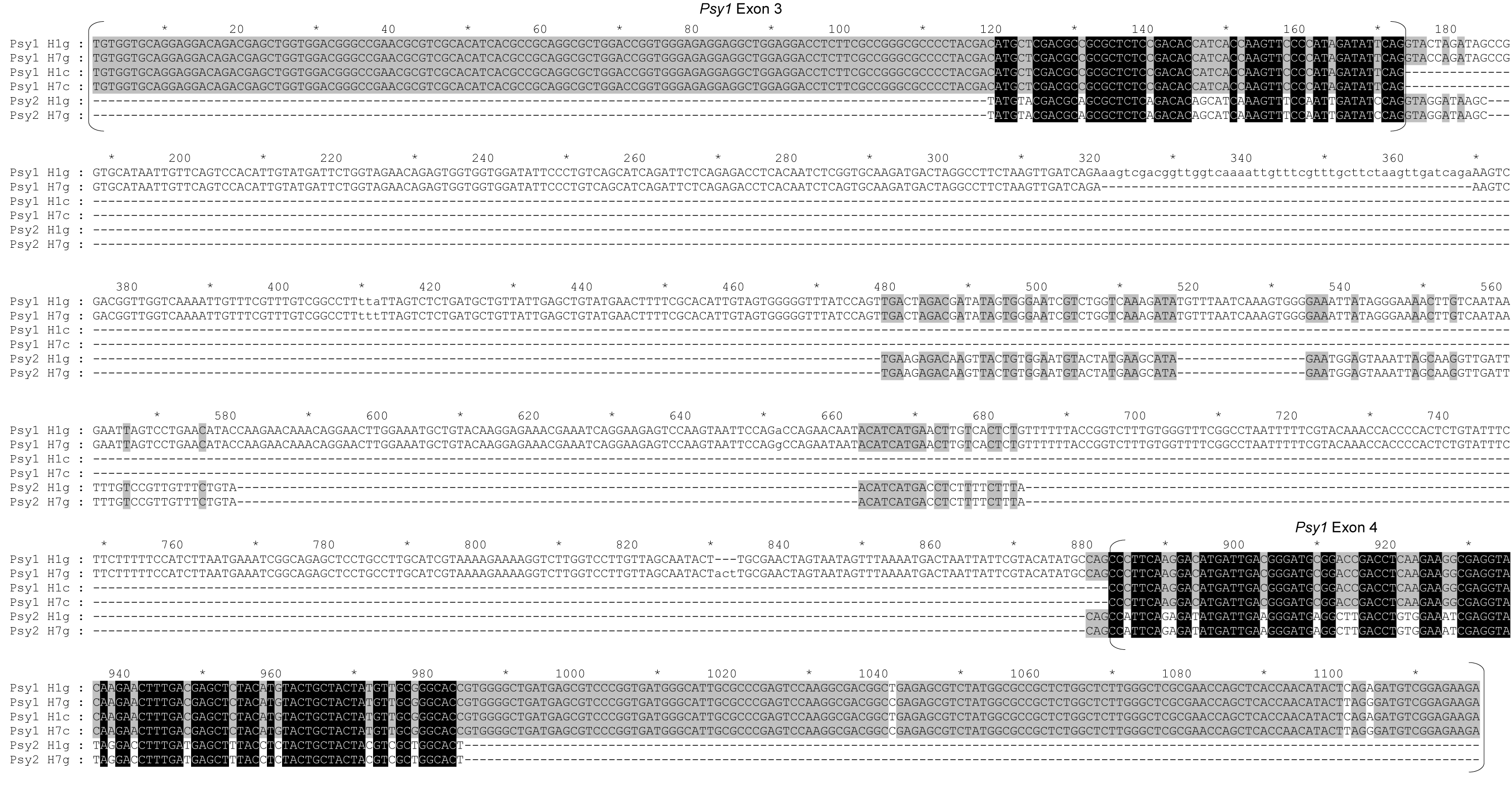

Supplement: Figure S4 — Alignment of partial Psy1 and Psy2 sequences in H1 and H7 lines of H. chilense. Alignment of genomic (g) and cDNA (c) sequences of Psy1 (this work) and partial genomic sequence of Psy2 gene in H1 and H7 lines (DU796678 and DU796680, respectively). (TIF) [file pone.0019885.s004.tif]

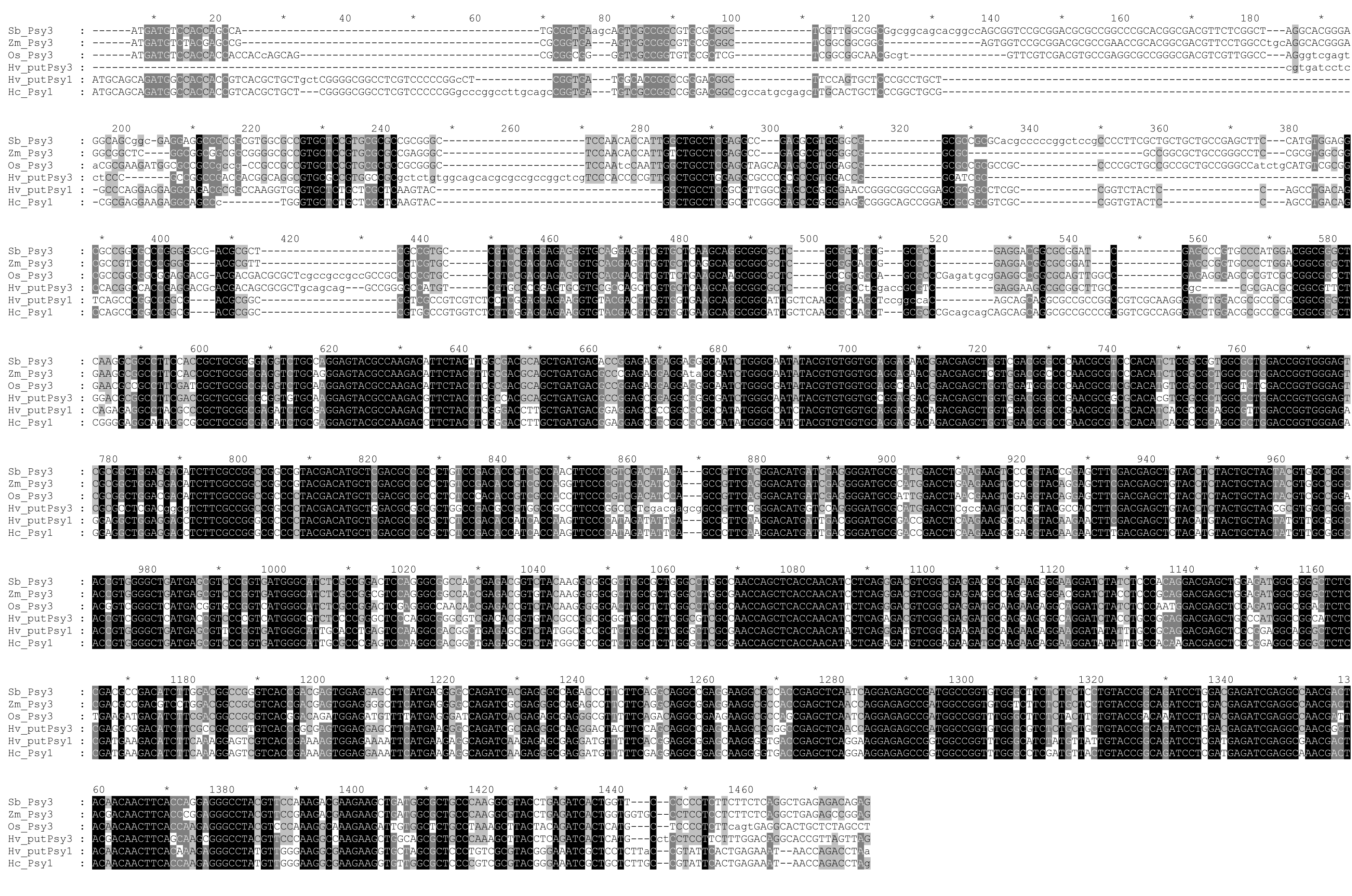

Supplement: Figure S5 — Alignment of Psy3 gene from different species. Alignment of Psy3 sequences of Sorghum bicolor (Sb; AY705390.1), Zea mays (Zm; NM_001114653.1), Oryza sativa (Os; FJ214953.1) and Hordeum vulgare clone NIASHv2034M16 showing high homology with Psy3 gene of other species (AK365521.1), Hordeum vulgare clone NIASHv3051L01 showing high homology with Psy1 gene of other species (AK374031.1) and H. chilense Psy1 gene (this work). (TIF) [file pone.0019885.s005.tif]
